# N6-methyladenosine participates in mouse hippocampus neurodegeneration via PD-1/PD-L1 pathway

**DOI:** 10.3389/fnins.2023.1145092

**Published:** 2023-05-10

**Authors:** Wen Hu, Hongbo Xie, Yubing Zeng, Pei Pei, Xiaojun Zhan, Shan Wang, Zhenlin Wang

**Affiliations:** ^1^Department of Otolaryngology-Head and Neck Surgery, Xuanwu Hospital, Capital Medical University, Beijing, China; ^2^Department of Otorhinolaryngology, Huadong Hospital Affiliated to Fudan University, Shanghai, China; ^3^Beijing Municipal Key Laboratory of Child Development and Nutriomics, Capital Institute of Pediatrics, Beijing, China; ^4^Department of Otorhinolaryngology Head and Neck Surgery, Children’s Hospital, Capital Institute of Pediatrics, Beijing, China

**Keywords:** m^6^A, hippocampus, neurodevelopment, aging, cognition, PD-1/PD-L1 pathway

## Abstract

Developmental abnormalities and hippocampal aging leads to alteration in cognition. In the brain, N6-methyladenosine (m^6^A) is a common and reversible mRNA alteration that is essential for both neurodevelopment and neurodegeneration. However, its function in the postnatal hippocampus and the specific mechanisms regulating hippocampus-related neurodegeneration still awaits elucidate. We identified dynamic m^6^A modifications in postnatal hippocampus at different stages (at 10 days postnatally, and at 11 and 64 weeks of age). m^6^A shows a definite cell-specific methylation profile and m^6^A modification displays temporal dynamic during neurodevelopment and aging. Differentially methylated transcripts in the aged (64-week-old) hippocampus were enriched in microglia. The PD-1/PD-L1 pathways was identified that may participate in the cognitive dysfunction associated with an aged hippocampus. Furthermore, Mettl3 was spatiotemporally expressed in the postnatal hippocampus, which was highly expressed at the age of 11 weeks compared with the other two timepoints. Ectopic expression of METTL3 in mice hippocampus mediated by lentiviral infection resulted in high expression of genes related to PD-1/PD-L1 pathway and significant spatial cognitive deficit. Together, our data show that m^6^A dysregulation, which is mediated by METTL3, most likely contributes to cognitive deficits linked to the hippocampus via the PD-1/PD-L1 pathway.

## 1. Introduction

Epigenetic regulation, which does not alter the DNA sequence, is a heritable and reversible modification of gene expression. N6-methyladenosine (m^6^A) is a recently discovered, abundant regulatory modification that dynamically mediates mRNA function (Meyer et al., 2012; Jia et al., 2013; Fu et al., 2014). m^6^A regulates RNA processes, including nuclear export (Roundtree et al., 2017), degradation (Oerum et al., 2021), splicing (Jiang et al., 2021; Mendel et al., 2021) and translation (Yu et al., 2018; Liu et al., 2020; Su et al., 2022). These dynamic effects are regulated by methylation transferases, demethylases, and m^6^A-binded proteins. Methyltransferases, also generally regarded as writers, allow m6A methylation to modify bases on mRNA. Methyltransferases such as METTL3, METTL14, and WTAP are core proteins of m^6^A methyltransferases (Ping et al., 2014; Cao et al., 2016). As well as in inflammatory responses (Liu et al., 2019; Wang et al., 2019) and immune responses (Wang et al., 2020), METTL3 is crucial for cell proliferation (Li et al., 2017), migration, invasion (Lin et al., 2016), apoptosis (Lin et al., 2019), and differentiation (Lin et al., 2017). The role of demethylation enzymes (“erasers”), the best known of which are FTO (Jia et al., 2011) and ALKHB5 (Zheng et al., 2013), demethylate bases that have been modified with m^6^A. m^6^A-binding proteins (“readers”), including proteins with the YTH structural domain (Wang et al., 2015; Li et al., 2017; Shi et al., 2017), can recognize m^6^A.

m^6^A RNA modifications are found throughout the brain and are responsible for a wide range of biological processes. Numerous studies have shown that m^6^A regulates brain function (Nainar et al., 2016; Xiong et al., 2021), axon regeneration (Weng et al., 2018; Zhang et al., 2021), brain development (Yoon et al., 2017; Wang et al., 2018), and learning and memory (Shi et al., 2018; Leonetti et al., 2020). Additionally, dysregulation of m6A modification has been linked to age-related neurodegenerative illnesses like Parkinson’s disease (Chen et al., 2019; Qin et al., 2020) and Alzheimer’s disease (AD; Zhao et al., 2021; Zhang et al., 2022), according to recent studies. In AD mouse models (Han et al., 2020; Shafik et al., 2021) and forebrain organoids (Yoon et al., 2017), signaling pathways linked to AD were enriched with differently methylated transcripts. In conclusion, a large body of research has established the regulatory function of m6A in age-related neurodegenerative disorders.

The hippocampus is important for learning, memory development, and memory consolidation. Abnormal development and aging of the hippocampus can lead to cognitive deficits. However, cognitive decline is not an inevitable consequence in early aging, and it is important to identify the neurobiological mechanisms that influence cognitive outcomes. Recent work suggests that epigenetics, especially DNA methylation and histone acetylation, influence cognitive ability across the lifespan (Day and Sweatt, 2011; Peixoto and Abel, 2013; Zovkic et al., 2013). However, the mechanisms underlying m^6^A’s regulatory involvement in hippocampus function in the context of normal aging remain to be elucidated.

**Figure 1:**
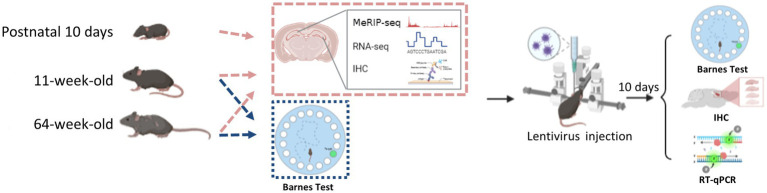
The flowchart of this study.

Here, we investigated how m^6^A’s dynamic regulatory actions affected the neurodevelopment of the mouse hippocampus and showed the biological importance of altered METTL3 expression. This work reveals the m^6^A plays a pivotal role in hippocampal neurodevelopment and aging; m^6^A methylation affects hippocampus-related cognition function through the PD-1/PD-L1 pathway. Genes associated with the PD-1/PD-L1 pathway experienced alterations in expression as a result of METTL3 knockdown. To provide a basis for exploring the molecular mechanisms of cognitive impairment associated with hippocampal aging (Figure 1).

## 2. Materials and methods

### 2.1. Animal

Male and female wild-type C57BL/6 mice, aged 10 days, 11 weeks, or 64 weeks, were used in this investigation. They were all obtained from Vital River Company (Beijing, China). Following the recommendations of Capital Medical University’s Animal Care and Use Committee, the mice were cared for, housed, and put to death.

### 2.2. Cell lines

The National Infrastructure of Cell Line Resource provided the Neuro2a (N2a) cells that were used in the *in vitro* transfection experiments (Beijing, China). The cells were grown in MEM medium (Gibco, 10,370,021), supplemented with 10% fetal bovine serum (Gibco, 10,099,141) and 1% penicillin–streptomycin (Gibco, 15,140,122). The manufacturer verified the cell lines and examined them for mycoplasma infection.

### 2.3. Barnes maze test

**Figure 2:**
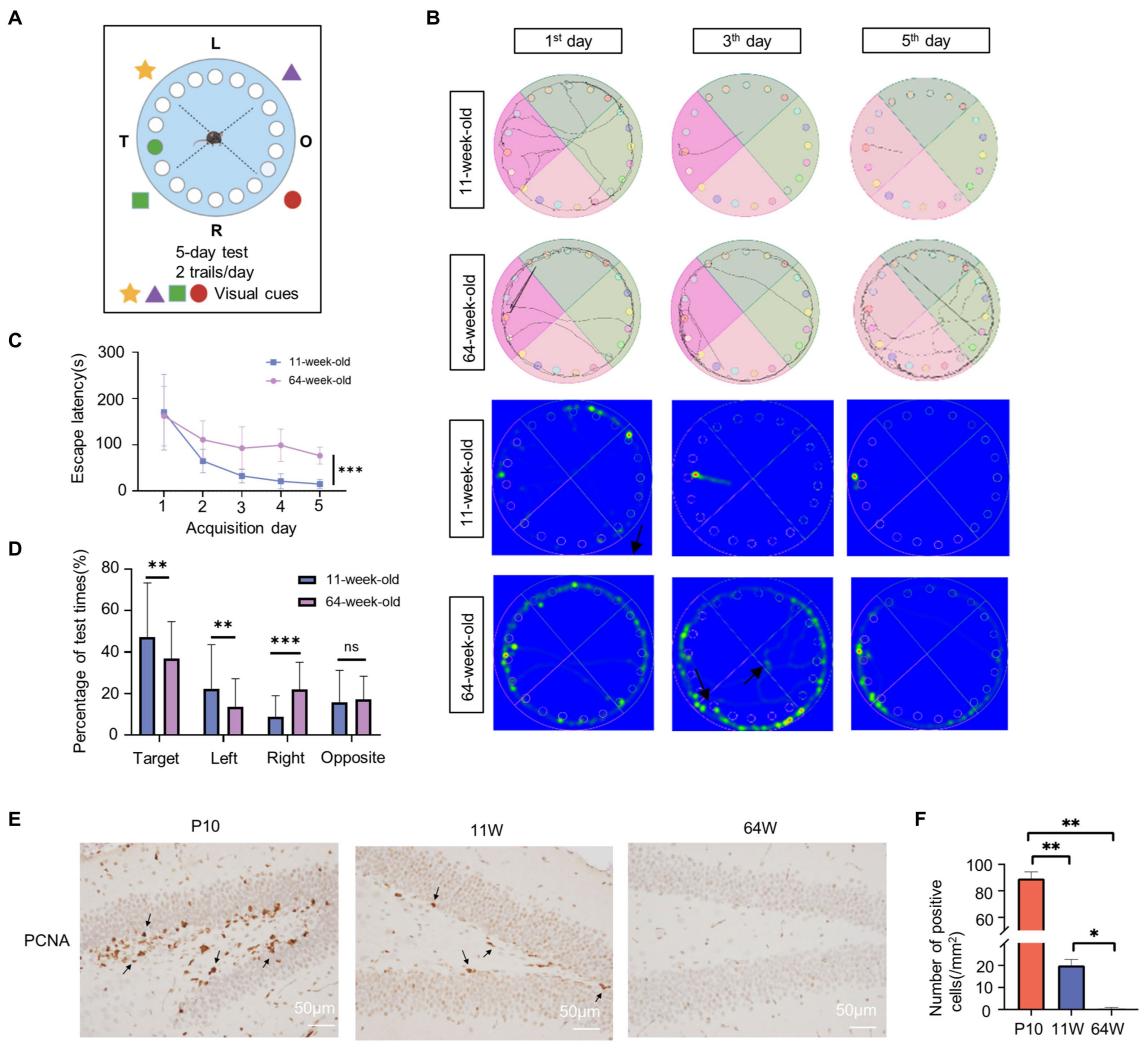
Age influence spatial cognition in mouse hippocampus. **(A)** Schematic diagram of Barnes experiment. **(B)** Tracking map and heat map of 11-week-old and 64-week-old mice in the Barnes maze. The represent tracking map showed the total distance traveled and the total time spent by the mice in the maze. In the heat map, the area with higher values of optical density indicated that the mice spent more time in that area. **(C)** Spatial learning curves in the Barnes Maze training sessions for 11-week-old and 64-week-old mice. (n = 8 per group, 4 female and 4 male) *t* = 7.55, df = 14, *F* = 2.204, ***p < 0.001, by Student’s t-test. **(D)** The quadrant time (%) exploring the four sectors was measured. (*n* = 8 per group, 4 female and 4 male), Target: *F* = 4.458, t = 2.673, df = 78, *p* = 0.009; Left: *F* = 1.873, *t* = 0.175, df = 78, *p* = 0.008; Right: *F* = 2.746, t = −4.366, df = 78, *p* = 0.000; Opposite: *F* = 3.951, t = 0.822, df = 78, *p* = 0.414; by statistical analysis by Student’s *t*-test. **(E,F)** Immunostaining of mouse hippocampus at postnatal 10 days, 11-week-old and 64-week-old using antibodies against the PCNA **(E)** and quantifications of positive cell per mm^2^ are shown as box plots **(F)**, with the horizontal lines representing the median. (*n* = 3). P10 vs. 11 W: *p* = 0.001; 11 W vs. 64 W: *p* = 0.013; P10 vs. 64 W: *p* = 0.003, by one-way ANOVA.

The Barnes maze test was performed according to previous reports with some modifications (Barnes, 1979; Patil et al., 2009; Rosenfeld and Ferguson, 2014; Nunes et al., 2015). The test involves a circular platform (90 cm in diameter) with 20 5-cm-diameter holes around the periphery. The holes are uniform in appearance, but only one is connected to a removable escape box that is 28 cm long, 22 cm wide, and 21 cm deep. Extra-maze cues throughout the maze serve as reference points that facilitate learning of the position of the target (escape) hole (Figure 2A). A video camera positioned immediately above the platform communicates with the Labmaze animal behavior analysis software (ver. 3.0; Zhongshi Technology, Beijing, China). A 500 W white-light bulb served as the platform escape stimulus in the pre-training trial, which involved placing each mouse in the center of the maze (in the start chamber). The chamber was taken out after 10 s, and the mouse was trained to use the escape hatch (by guiding it toward the box, in which it remained for 4 min). Each trial began with the mouse being confined to the same start chamber; 10 s after light onset, the chamber was removed, and the mouse was then free to explore the maze. The experimenter observed the mouse from behind a curtain. When all four limbs of the mouse entered the target hole, the behavior was counted as an escape. The mouse was allowed to enter the escape box and stay within for 30s. In each trial, each animal was observed for up to 4 min. The animal was gently steered toward the hole leading to the escape box if it did not reach the target hole in 4 min. The mouse was then removed from the experiment and put back in its cage for a 15-min rest before the next trial began. To avoid the usage of intra-maze signals, the platform was cleaned with a 70% alcohol solution after each trial. All mice were trained twice a day for 5 days. The distance traveled and the amount of time spent in the target zone, as well as the latency to recognize and enter the escape box, were all recorded.

### 2.4. Immunohistochemistry and immunofluorescence analysis

Mice were injected with 4% paraformaldehyde following anesthesia. Whole brains were extracted and post-fixed for 48 h at 4°C in 4% paraformaldehyde. The brains were next phosphate-buffered saline washed, ethanol dehydrated, and paraffin embedded. For the immunohistochemistry study, we employed 4-m-thick slices. The main antibodies METTL3 (1:50 dilution; Cell Signaling Technology [CST], United States), GFP antibody (1:500 dilution; Abcam, United States), NeuN (1:400 dilution; CST, USA), DCX (1:400 dilution; CST, United States), and Iba1/AIF-1 were employed for incubation at 4°C overnight (1:800 dilution; CST). To check the quality of the slides, a negative control was included in each batch of slides that used 10% regular goat serum instead of the primary antibody. By using optical density analysis, the levels of target protein were identified. For immunofluorescence, secondary antibodies conjugated to Alexa Fluor 488 or Alexa Fluor 594 (1:200 dilution; Zhongshan Jinqiao Biotechnology Co., Beijing, China) were used to see the primary antibodies.

### 2.5. Reverse transcription and quantitative real-time polymerase chain reaction

ABM, Richmond, Canada’s Revert Aid First Strand cDNA Synthesis Kit was used to create cDNA from the total RNA that was isolated from the mouse hippocampus using the TRIzol reagent (Invitrogen, Waltham, MA, United States). Real-time polymerase chain reaction (RT-qPCR) was used to examine the cDNA in order to ascertain the relative RNA levels of the target genes using the Maxima SYBR Green/ROX qPCR Master Mix (ABM). Following is the RT-qPCR process: one cycle of 95°C for 10 min., 35 cycles of 95°C for 35 s and 60°C for 60 s. The following primers were used:

Mouse *METTL3* forward:5′-CGCTGCCTCCGATGTTGATCTG-3′.
Mouse *METTL3* reverse:5′-TCTCCTGACTGACCTTCTTFCTCTG-3′;
Mouse *Pdcd1* forward:5′-AGGGTGTACAGGCTCCTTCC-3′.
Mouse *Pdcd1* reverse: 5′-AGAAGGTGAGGGACCTCCAG-3′.
Mouse *Myd88* forward: 5′-GAGGAGGAGCCTCTACACCC-3′.
Mouse *Myd88* reverse: 5′-ACACTGCTTTCCACTCTGGC-3′.
Mouse *Ptpn6* forward: 5′-CAGAAGAGGAGGGGCAAAGA-3′.
Mouse *Ptpn6* reverse: 5′-GAGGTGGGAGCTAAACAGGT-3′.

### 2.6. Western blot

The primary antibody, a mouse anti-METTL3 monoclonal antibody (1:1,000; Proteintech), was incubated on the blots for an entire night at 4°C before the secondary anti-rabbit HRP-conjugated antibody (1:5,000; CST) was applied for an hour at room temperature. Thermo Fisher Scientific, Waltham, Massachusetts, United States, generated the blots with SuperSignal West Pico Chemiluminescence Substrate, and Quantity One software was used to quantify the results using a densitometer (Universal Hood II; Bio-Rad, Hercules, California, United States).

### 2.7. Plasmid construction

This study used the lentiviral knockdown system composed of pLV3-CMV-GFP-Puro, PG-P1-VSVG, PG-P2-REV, and PG-P3-RRE. In the LV3 vector, mouse METTL3 shRNA was inserted between the HIV 3 LTR and the H1 promoter. Transducing 293 T cells was used to titrate the lentivirus (tenfold serial dilution). The lentivirus utilized in this investigation typically had a titer between 1,108 and 11,010 TU/ml. In N2a cells, the effectiveness of the knockdown was assessed by Western blot. The following oligonucleotide sequences were used for METTL3 shRNA: mouse METTL3-shRNA: 5′-GCACACTGATGAATCTTTAGG-3′; and scramble control: 5′-TTCTCCGAACGTGTCACGT-3′.

### 2.8. Lentivirus infection of mouse hippocampus

The injection was performed according to previous reports (Shi et al., 2018). The male 11-week-old mice were put on a stereotaxic device after being given isoflurane anesthesia. Isoflurane concentration of 4% for induction of anesthesia in mice and 1.2% for maintenance of anesthesia during lentiviral injection. A micro syringe was used to sequentially inject polybrene (5 g/ml) and lentivirus (1 μl) into either side of the hippocampus (− 2 mm anterior/posterior (A/P), 1.5 mm medial/lateral (M/L), 1.6 mm dorsal/ventral (D/V) from bregma) at a rate of 0.25 μl per minute. Five minutes after the lentivirus infusion, the injection syringe was gradually removed. At 37°C, mice were brought back to their original cages after being awakened from anesthesia. At least 10 days after the virus injection, behavioral tests or other types of analysis were carried out. All behavioral tests were finished with an examination of lentivirus infection by RT-qPCR or immunohistochemical analysis.

### 2.9. RNA isolation, m^6^A-IP, m^6^A-seq, and MeRIP-qPCR

In order to dissect the hippocampus, mice were killed through cervical dislocation. There were three biological replications. Five mice hippocampus were merged into a single sample, and each set of 15 animals underwent three rounds of MeRIP-seq analysis. Shanghai Cloud-seq Biotech Co. finished MeRIP-seq. TRIzol was used to purify total RNA (Invitrogen). Each sample’s RNA concentration was calculated using the Thermo Fisher Scientific NanoDropND-1,000 instrument, and samples with OD260/OD280 ratios of 1.9–2.1 were used in subsequent tests. Denatured agarose gel electrophoresis was used to check the RNA integrity and gnomic DNA contamination.

The m^6^A-IP Kit (GenSeq Inc., Shanghai, China), in accordance with the manufacturer’s instructions, was used to IP total RNA. Utilizing RNA Fragmentation Reagents, RNA was randomly fragmented to a length of 200 nt (Thermo Fisher Scientific). Rotational coupling of Protein A/G beads to the m^6^A antibody was carried out for 1 h at room temperature. The bead-linked antibodies were incubated with the RNA fragments and rotated at 4°C for 4 h. The trapped RNA was eluted from the complexes and purified after the RNA/antibody complexes had been incubated. The Low Input Whole RNA Library Prep Kit (GenSeq.) was then used to create RNA libraries for IP and input samples, according to the kit’s instructions. Libraries were qualified using the 2,100 Bioanalyzer instrument (Agilent Technologies, Santa Clara, CA, United States) and then sequenced using the NovaSeq platform (Illumina, San Diego, CA, United States). In Supplementary Table S1, the quality assurance metrics for the sequencing data are presented.

Three differentially methylated RNA sites were selected to design specific primers for MeRIP-qPCR using NCBI Primer-Blast (Ye et al., 2012). Sangon Biotech Co. produced the forward and reverse primers (Shanghai, China). Following that, qPCR was carried out utilizing the QuantStudioTM 5 System and PrimeScript RT Reagent Kit (both from Takara, Shiga, Japan) to produce cDNA from reverse-transcribed IP RNA (Thermo Fisher Scientific).

### 2.10. RNA sequencing

Hippocampus samples from five mice were combined into one sample and 15 hippocampus were used in total to conduct three biological replicates. The rRNAs were removed from total RNA using the rRNA Removal Kit (GenSeq). Then, the rRNA-depleted samples were subjected to library construction using the Low Input RNA Library Prep Kit (GenSeq) according to the manufacturer’s instructions. Libraries were subjected to quality control and quantified using the BioAnalyzer 2,100 system (Agilent Technologies). Library sequencing was performed using the Illumina NovaSeq instrument with 150-bp paired end reads.

### 2.11. m^6^A peak calling, differential peak calling, and motif analysis

Paired-end reads were harvested using the NovaSeq 6,000 sequencer (Illumina) and quality controlled based on the Q30 score. Clean reads from all libraries were aligned to the reference genome (mm10) using Hisat2 software after 3′ adaptor-trimming and low-quality read removal using cutadapt software (v1.9.3).

Clean reads from all libraries were aligned to the reference genome (mm10) using Hisat2 software (v2.0.4; Anders et al., 2015), after 3′ adaptor-trimming and low-quality read removal using cutadapt software (v1.9.3; Martin, 2011). MACS program was used to find the methylated sites on RNAs (peaks; Zhang et al., 2008). DiffReps detected differentially methylated sites (Shen et al., 2013). Peaks identified by both software packages that overlapped with exons of mRNA were identified and selected using in-house scripts. MEME software (version 5.1.1; https://meme-suite.org/meme/) was used to search for motifs enriched in m^6^A peaks. Heatmaps of the *p*-values of motifs were generated using the pheatmap R package. To view m6A peaks throughout the whole transcript, Integrative Genomics Viewer^1^ was employed.

### 2.12. Gene ontology, kyoto encyclopedia of genes and genomes and cell marker analyses

ClusterProfiler (ver. 4.2.2; https://github.com/YuLab-SMU/clusterProfiler) was used to perform Gene Ontology (GO) enrichment analysis, Kyoto Encyclopedia of Genes and Genomes (KEGG) analysis, and cell marker analysis. GO/KEGG terms and cell markers were used to create plots using the ggplot2 R package (Ginestet, 2011).

### 2.13. Quantification and statistical analysis

All the experiments were repeated independently at least three times, and the data were expressed by mean ± SD. Barens test results were analyzed using a multivariate ANOVA. Two and more than two groups were using Student’s test and one-way ANOVA, respectively. A *p* value of <0.05 was statistically significant and is presented as *p < 0.05, **p < 0.01, or ***p < 0.001.

## 3. Results

### 3.1. Age-related changes in spatial cognition

The hippocampus is a critical structure for learning and memory; it is in charge of memory formation as well as spatial orientation. P10, 11-week-old, and 64-week-old mice were used to represent the neurodevelopment, adult, and aging stages, respectively. Since mice exhibit rapid development at P10, at which time their motor and spatial cognitive functions are not fully established, we only used the Barnes maze test on 11- and 64-week-old mice to examine changes related to age in spatial cognition (Figure 2A). As expected, all parameters assessed during the test showed a marked decline in the 64-week-old mice. The two groups both performed a high variety at the first trial day. As the test progressed, 64-week-old mice required more time to be consistent in performance compared to 11-week-old mice. These indicate that older mice are poorer in the learning compared to the 11-week-old mice. And the 11-week-old mice exhibited clear improvement in spatial memory; the distance traveled, and number of error hole probes decreased significantly in these mice (Figure 2B). 64-week-old mice had considerably longer escape latency than 11-week-old mice (*F* = 2.38, *p*<0.000; Figure 2C). There was no significant difference between male and female mice in each group (11-week-old: *F* = 4.751, *p* = 0.632; 64-week-old: *F* = 2.672, *p* = 0.089) (Supplementary Figure S1A). Age had significant effect on the escape latency time (*F* = 64.638, df = 1, *p* = 0.000), while sex and sex × age interaction was not significant for latency time (Supplementary Table S2). In addition, the proportion of time spent exploring the target area increased in both groups. However, the proportion of time spent in the target area was significantly higher in the 11-week-old mice (Target: *F* = 4.458, *p* = 0.009; Left: *F* = 1.873, *p* = 0.008; Right: *F* = 2.746, *p*<0.000; Opposite: *F* = 3.951, *p* = 0.414) (Figure 2D; Supplementary Table S2). The quadrant time spend was affected by age, sex, and quadrant (age: *F* = 4.398, *p* = 0.037; sex: *F* = 4.248, *p* = 0.04; quadrant: *F* = 32.128, *p*<0.000) (Supplementary Table S2). The 11-week-old male mice performed better spatial cognition and memory. However, there is no significant difference in sex within group (Supplementary Figure S1B). The age×quadrant interaction was significant for quadrant time spent (*F* = 8.553, df = 3, *p*<0.000; Supplementary Table S2). Overall, the results indicated learning and cognitive deficits in the 64-week-old mice. In addition, we determined cell proliferation in the mice hippocampus of the three stages by immunohistochemistry to identify age-related changes in the hippocampus (Figures 2E,F). The PCNA-positive cells in the 16-month-old hippocampus were almost zero, suggesting that the cell proliferation capacity of the hippocampus at this stage was significantly reduced.

### 3.2. Landscape of m^6^A methylation in mouse hippocampus from neurodevelopment to aging

**Figure 3:**
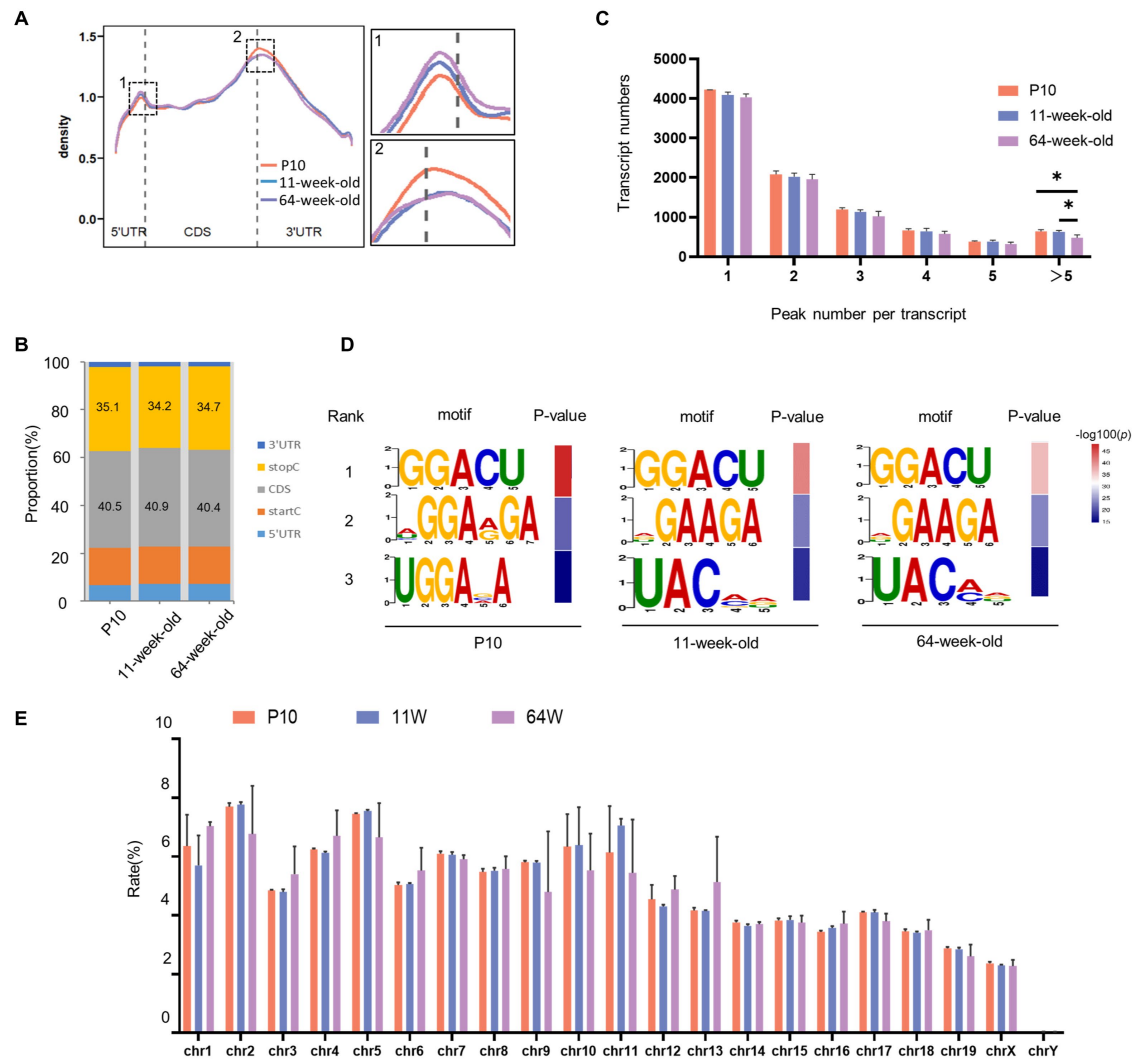
Overview of m^6^A modified transcripts in the hippocampus of mouse at three periods. **(A)** Positions of detected m6A peaks on all methylated transcripts in hippocampus were determined. **(B)** The distribution of m^6^A peaks in three stages, respectively. **(C)** Numbers of transcripts containing different numbers of m^6^A peaks per transcript from P10 to 64 weeks old. (11 W vs. P10: *p* = 0.047;64 W vs. 11 W: *p* = 0.033, by one-way ANOVA). **(D)** The top three motif sequences for m^6^A-containing peaks in P10, 11 W and 64 W. **(E)** The count of m^6^A peaks in mouse chromosomes.

To comprehend the biological significance of m6A in the hippocampus throughout the lifespan, we profiled the m^6^A transcriptome using MeRIP-seq at the three time points (P10, 11 weeks and 64 weeks; Supplementary Table S1). GUITAR graphs summarizing the priority region and average distribution of m^6^A peaks in the hippocampus (Figures 3A,B) revealed that the peaks were predominantly found in the coding sequence and 3′ UTR of mRNA. From P10 to 64 weeks, most transcripts contained only one m^6^A peak (Figure 3C). The number of transcripts containing >5 peaks was significantly lower in the 64-week-old group than in the P10 and 11-week-old groups. We performed motif searches of all detected peaks. In accordance with earlier research (Dominissini et al., 2012; Meyer et al., 2012), the m^6^A peaks were mostly characterized by GGACU in all three age groups. In addition, the expressed m^6^A peaks were found on all chromosomes, but mainly on chrome 1, chrome 2, chrome 5, and chrome 11 (Figure 3E).

### 3.3. m^6^A Methylation-enriched genes are involved in important biological processes in the hippocampus across the lifespan

**Figure 4:**
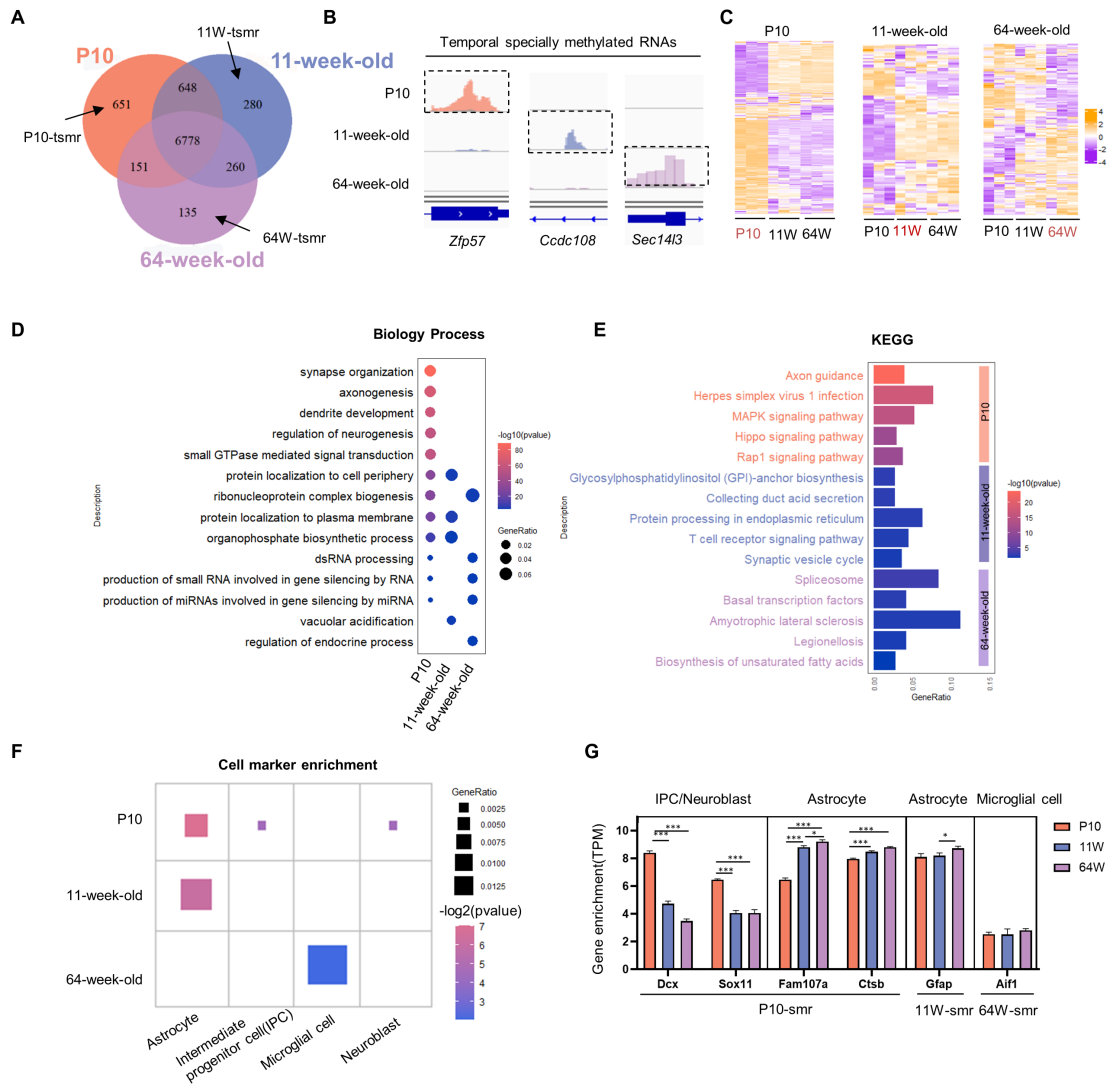
Characterizing temporal-specific m^6^A methylation across lifespan of the hippocampus. **(A)** Venn diagram showing the numbers and relationship of methylated gene at three stages. **(B)** Integrative Genomics Viewer (IGV) plots showing examples of temporal-specific methylated genes. Colored reads originate from m^6^A-IP libraries. The number of read counts was normalized among three groups per gene. Dashed boxes indicate the position of temporal-specific m^6^A peaks. **(C)** Heatmaps showing the correlation between mRNA expression levels and temporal specially methylation at three stages, respectively. **(D)** Distribution of GO biological process terms significantly enriched for TSMRs in the three stages. **(E)** Significantly enriched pathways for TSMRs. **(F)** Cell marker analysis of enriched cell types of TSMRs in the three stages. **(G)** The comparison of expression value (TPM) of cell marker gene with temporal specifically methylation among three groups. *p < 0.05, ***p < 0.001, by one-way ANOVA.

To understand the temporal effect of m^6^A methylation across the lifespan, we analyzed the methylation profiles of the P10, 11-week-old mice, and 64-week-old mice; 8,228, 7,966, and 7,324 methylated poly(A) RNAs were identified, respectively (Figure 4A). We identified 6,778 RNAs continuously methylated throughout the lifespan, together with 651, 135, and 280 transcripts harboring RNAs methylated specifically at P10, 11 weeks, and 64 weeks, respectively (Figure 4B). At P10, the mRNA expression patterns of these “temporal specifically methylated RNAs” (TSMRs) were the exact opposite of those at 11 and 64 weeks (Figure 4C). Interestingly, the overall mRNA expression of the TSMRs at 11 weeks was elevated compared with the former stage but was similar to that at 64 weeks. This indicates that the regulation of mRNA expression of TSMRs differs among neurodevelopmental stages, although the adult and aging stages showed similarities.

To clarify the biological functions and signaling pathways of temporally specific methylation, we conducted GO and KEGG analysis of genes showing this type of methylation (Figures 4D,E). The TSMRs in all three age groups were annotated. Transcripts with P10-specific methylation were enriched in processes including the regulation of synaptic transmission, axon growth, and neurogenesis. These transcripts were consequently enriched in signaling pathways involved in nervous system development, including the axon guidance pathway and the MAPK signaling pathway. At 64 weeks, specifically methylated transcripts were enriched in the spliceosome pathway. These transcripts were connected to biological functions, such as the control of gene silence.

To identify the types of cells in the hippocampus that the TSMRs were enriched in, we performed cell marker analysis with clusterProfiler (Zhang et al., 2019; Wu et al., 2021). In accordance with their varying biological functions, the TSMRs were also associated with distinct cell types (Figure 4F). At P10, temporally specific methylation was noted for cell marker genes associated with IPCs, neuroblasts and astrocytes. At 11 weeks, TSMRs were enriched in astrocytes, which maintain neuronal function, while at 64 weeks there was enrichment in microglial cells, which are implicated have been linked to the onset and development of many neurological diseases. Next, we compared the expression of cell marker genes showing temporally specific methylation among the three stages (Figure 4G). Although TSMRs were enriched in astrocytes at both P10 and 11 weeks, the enriched cell marker genes were different. At the P10 stage, enrichment was mainly seen for genes involved in cell proliferation and cell cycle progression. In contrast, at 11 weeks old, TSMRs were mainly enriched in cell marker genes of mature astrocytes. At 64 weeks, old TSMRs were enriched in microglial cells. Some studies have indicated that microglia linked to the progression of AD, which is an age-related condition (Sarlus and Heneka, 2017; Olah et al., 2020).

Taken together, the data suggest that m^6^A differentially affects mRNAs to ensure proper hippocampal development across the lifespan.

### 3.4. m^6^A methylation regulate transcription in the hippocampus across a lifespan

**Figure 5:**
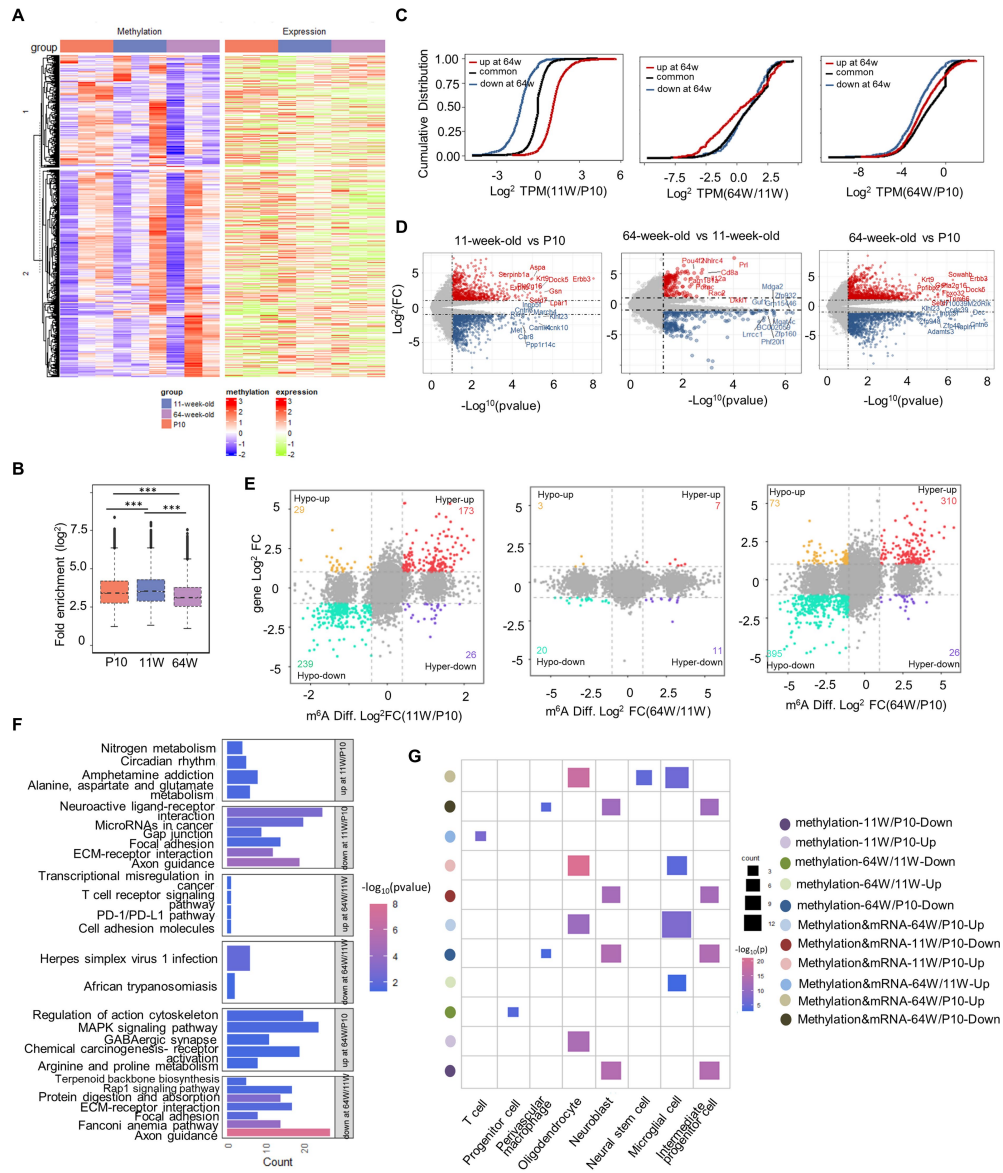
Differential methylation and mRNA expression profiles in the hippocampus. **(A)** Heatmap correlating mRNA methylation levels (Fold Enrichment) and mRNA expression levels (TPM) in mouse hippocampus from P10 to 64 weeks old. **(B)** Box plot showing the relative methylation levels of P10, 11 weeks old and 64 weeks old as evaluated by the fold enrichment of all m^6^A peaks. ***p < 0.001, by one-way ANOVA. **(C)** Cumulative frequency plots showing how methylation status correlates with mRNA expression levels between P10, 11 weeks old, and 64 weeks old hippocampus pairwise comparisons. **(D)** volcano plots showing the DEGs between pairwise comparisons (|Fold Change| ≥ 2, *p-*value < 0.05). **(E)** Nine quadrant graphs exhibiting the DEGs containing differently methylation. **(F)** KEGG analysis of enriched pathways of differentially methylated genes that are upregulated or downregulated between pairwise comparisons. **(G)** Cell marker analysis of enriched cell types of differentially methylated genes that are hypomethylated or hypermethylated and DEGs containing differently methylation that are upregulated or downregulated between pairwise comparisons.

As m^6^A is temporally regulated in the mouse hippocampus postnatally, we next compared the methylated gene profiles among the three stages. The number of fold-changes of m^6^A peaks in the hippocampus increased at 11 weeks compared with P10 (Figure 5B), and the number of fold-changes was lowest at 64 weeks. Next, we identified significantly hyper- and hypomethylated mRNAs and performed pairwise comparisons between two stages (Supplementary Figure S2A). Significantly differentially methylated genes (DMGs) were subjected to GO and KEGG pathway analysis to investigate the physiological roles of m6A alterations throughout the three life stages. The top 10 enriched biological processes of the DMGs were identified (Supplementary Figure S2B). At P10, the DMGs were mainly involved in axon development. In contrast, the DMGs at 64 weeks were mainly associated with biological processes such as ion transport, neurotransmission, and ATP metabolism. The KEGG pathway analysis revealed that DMGs at P10 were involved in pathways related to neuronal development, while those at 64 weeks were significantly associated with oxidative phosphorylation, which is a metabolic change implicated in aging and immunity (Mittelbrunn and Kroemer, 2021; Møller et al., 2022; Supplementary Figure S2C).

Given the critical function of m6A alterations in controlling gene expression, we examined the relationship between m6A and mRNA levels (Figure 5A). Differentially methylated transcripts exhibited significant differences in mRNA expression among the life stages (Figure 5C). Transcripts that were differentially methylated at 11 weeks usually exhibited significantly different mRNA expression levels among life stages than those showing no change in methylation from P10 to 11 weeks. However, by 64 weeks, the effect of differential methylation on mRNA expression was less pronounced. To gain understanding of the changes in epitranscriptome-wide among the three groups of methylated mRNAs, we identified differentially expressed genes (DEGs) at both the transcriptional and methylation levels, and then performed pairwise comparisons (Figures 5D,E). Few DEGs and DMGs were found between the 11- and 64-week-old groups. To better understand which types of cells in mouse hippocampal signaling pathways these genes are associated with, we performed KEGG and cell marker enrichment analyses (Figures 5F,G). At P10, simultaneous differential methylation and expression was detected in many genes involved in axon guidance and the extracellular matrix-receptor interaction pathway. These genes were enriched in neural stem cells, neuroblasts, and IPCs. Interestingly, DEGS between 64 weeks and the other life stages were enriched in the PD-1/PD-L1 checkpoint pathway. Finally, cell marker enrichment analysis revealed that enrichment was greatest in microglial and T cells, which play a role in the PD-1/PD-L1 checkpoint pathway. These results suggested that m^6^A methylation likely modulates changes in cognitive function related to hippocampal aging through the PD-1/PD-L1 pathway.

### 3.5. m^6^A methylation and mRNA expression of genes related to the PD-1/PD-L1 pathway increased in aging mice

**Figure 6:**
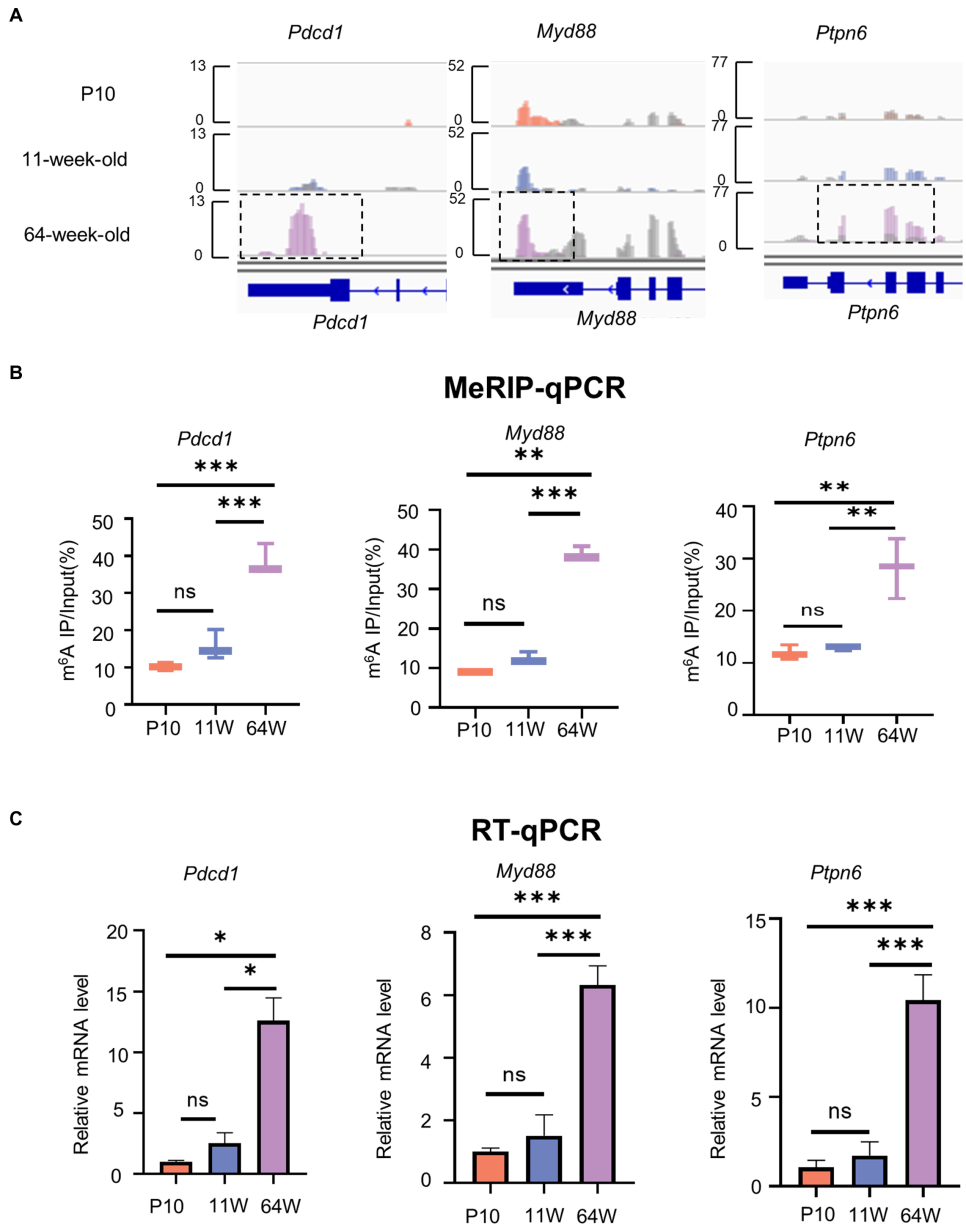
The level of m^6^A methylation and mRNA expression of genes related to PD-1/PD-L1 pathway are altered. **(A)** IGV plots showing methylated genes (*Pdcd1, Myd88,* and *Ptpn6*). Colored reads originate from m^6^A-IP libraries. The number of read counts was normalized among three groups per gene. Dashed boxes indicate the position of increased level of m^6^A peaks. **(B)** MeRIP-qPCR assay indicating the m^6^A methylation levels of PD-1/PD-L1 pathway-related genes Pdcd1, Myd88, and Ptpn6. **(C)** PD-1/PD-L1 pathway-related genes Pdcd1, Myd88, and Ptpn6 mRNA relative expression level in hippocampus from P10 to 64 weeks old were measured by RT-qPCR. Data are mean ± SD. (*n* = 3) **p < 0.01, ***p < 0.001, by one-way ANOVA.

PD-1 functions as a negative regulator of aging-related immune responses (Lages et al., 2010; Hong et al., 2019). We found that the m^6^A methylation of *Pdcd1*, *Myd88*, and *Ptpn6* mRNA, which are involved in the PD-1/PD-L1 pathway, was obviously increased in the 64-week-old mice (Figure 6A). To further verify the methylation levels of these three mRNAs, MeRIP-qPCR primers were designed (Table 1). MeRIP-qPCR confirmed the MeRIP-seq results, i.e., demonstrated increased m^6^A methylation levels of the three PD-1/PD-L1 pathway-related mRNAs in the 64-week-old hippocampus (Figure 6B; Supplementary Table S2). Furthermore, the three genes were upregulated in the 64-week-old hippocampus (Figure 6C; Supplementary Table S2). These findings suggested that m6A modification may play a regulatory role in the expression of *Pdcd1*, *Myd88*, and *Ptpn6* in the PD-1/PD-L1 pathway in the aging hippocampus.

**Table 1:** List of primers for MeRIP-qPCR used in this study.

Gene name	Primer sequencing
Ptpn6	Forward: GGGGCTAGACTGTGACATTG
	Reverse: TTCGATGAACTGGGCAATGG
Myd88	Forward: CCCTGAGTCCCCAAGAAAGT
	Reverse: GTGAAATGCCCCATGAGACC
*Pdcd1*	Forward: GGCTTCCCGGTTTCCTATTG
	Reverse: AGTCCCTAGAAGTGCCCAAC

### 3.6. Cognitive impairment result from Mettl3 knockdown in the hippocampus

**Figure 7:**
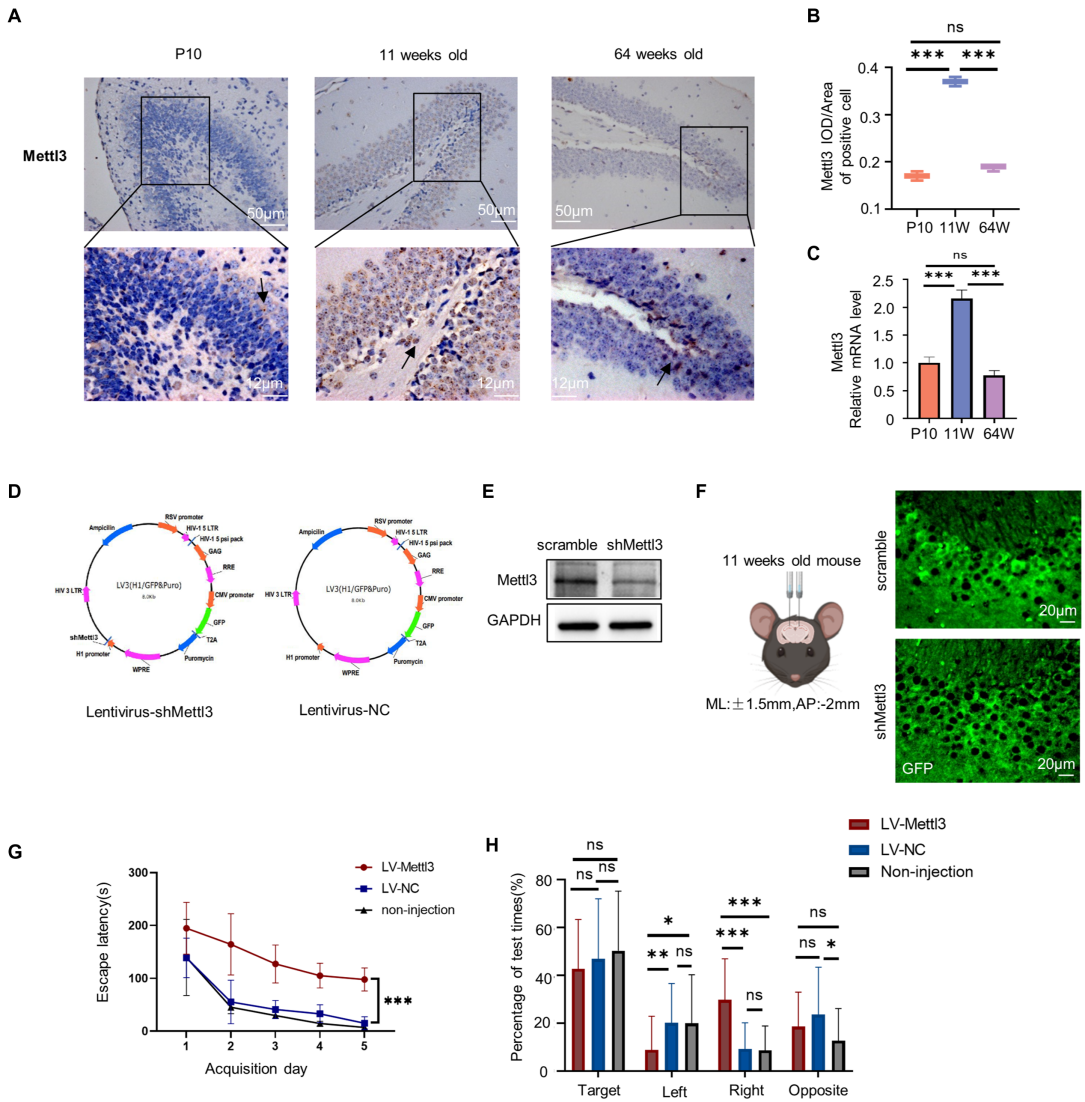
Spatial cognition alteration after infected with lentivirus for Mettl3 knockdown in the hippocampus. **(A,B)** Immunostaining of mouse hippocampus at postnatal 10 days, 11-week-old, and 64-week-old using antibodies against the Mettl3 **(A)** and quantifications of Mettl3 IOD/Area of positive cell are shown as box plots **(B)**, with the horizontal lines representing the median (*n* = 3). ***p<0.001, ns not significant, by one-way ANOVA. Hematoxylin staining were blue in the hippocampus. Mettl3-positively stained were brown. **(C)** Relative mRNA expression of Mettl3 in P10, 11 weeks old and 64 weeks old by RT-qPCR (*n* = 3). ***p<0.001, ns not significant, by one-way ANOVA. **(D)** Schematics of lentivirus vector constructs knockdown Mettl3 (Lentivirus-shMettl3) or control (Lentivirus-NC). **(E)** Western blot analysis to confirm the knockdown efficiency of Mettl3 in the N2a cells. **(F)** Illustration of lentivirus injections into the mouse hippocampus and images of GFP immunostaining (green) in an 11-week-old mouse injected with lentivirus after 10 days. **(G)** Spatial learning curves in the Barnes Maze training sessions for shMettl3 and control mice. (*n* = 7 per group) ***p<0.001, by Student’s *t*-test. **(H)** The quadrant time (%) exploring the four sectors was measured (*n* = 7 per group), *p<0.05, ns not significant, by Student’s *t*-test.

As a prerequisite of m^6^A modifications, we investigated the expression of the methyltransferase METTL3, which is well-known to play significant roles in neurodevelopment and neurodegeneration (Zhao et al., 2021). We applied immunohistochemistry to detect METTL3 protein expression *in situ* and confirmed that the expression level of METTL3 increased in the granular layer of the 11-week-old mouse hippocampus (Figures 7A,B; Supplementary Table S2). At the P10 stage, few METTL3-positive cells were seen in the dentate gyrus’s outer layer. The METTL3 level decreased again by 64 weeks of age.

We also performed RT-qPCR to further measure the *Mettl3* mRNA level, and observed similar trends (Figure 7C; Supplementary Table S2). The elevated *Mettl3* expression level at 11 weeks indicates that it plays an important role in this life stage. To confirm this, we altered the m^6^A levels in the 11-week-old mouse hippocampus by knocking down *Mettl3* using lentivirus plasmid (Figure 7D). Western blot analysis was conducted to evaluate the knockdown efficiency of shMettl3 in N2a cells *in vitro* (Figure 7E). Lentivirus were injected into hippocampus via stereotactic. We evaluated hippocampus-dependent spatial learning and memory using the Barnes maze test in these infected mice at least 10 days after virus injection (Figure 7F). And we confirmed the downregulation of Mettl3 expression level in shMettl3-injected mice hippocampus compared to the controls (Supplementary Figure S3A–E). *Mettl3* knockdown mice took longer to navigate and escape the maze than control mice (Figures 7G,H; Supplementary Table S2), indicating impairments in spatial learning and memory. The main effect of quadrant was significant in the probe test (*F* = 72.528, df = 3, *p*<0.000, Supplementary Table S2). The three group all spent more time in the target quadrant. The quadrant ×group interaction was also significant for quadrant spend time (*F* = 8.623, df = 6, *p*<0.000, Supplementary Table S2). For the target quadrant, the three group have no significant difference (Figure 7H). shMettl3-injected mice suffered a cognition impair and performed worse mainly in the escape latency time. These results suggest that appropriate *Mettl3* levels are crucial for spatial cognition. However, there was no significant difference between the control group and the noninjected mice, indicating that the injection of lentivirus in the hippocampus had no effect on hippocampal-related cognitive function. The cognitive deficits in the Mettl3 knockdown group were due to a single factor, Mettl3.

### 3.7. Mettl3 Knockdown induces neurodegeneration and mRNA changes of genes related to the PD-1/PD-L1 pathway

**Figure 8:**
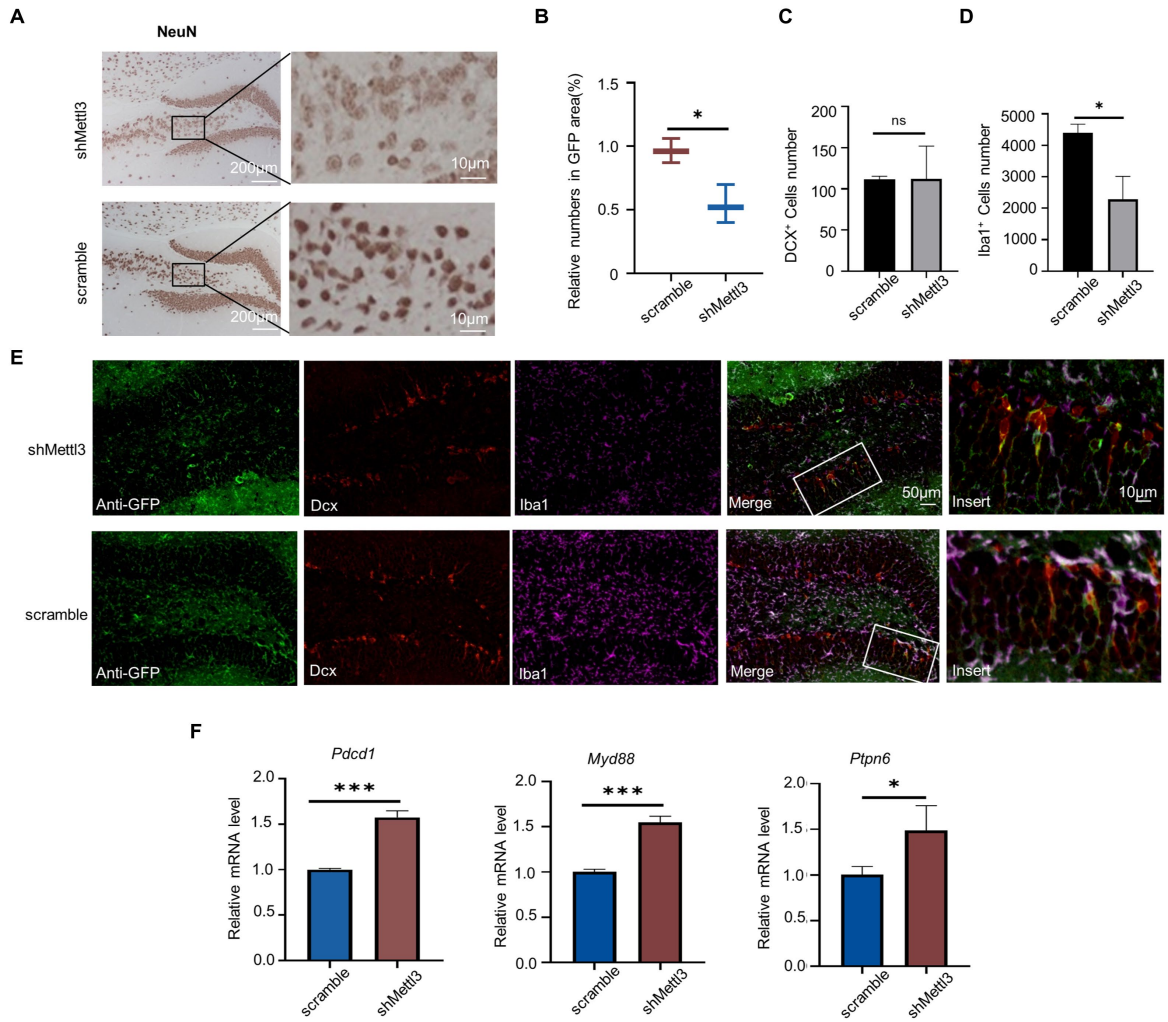
Mettl3 knockdown in hippocampus affect mature neuron and PD-1/PD-L1 pathway. **(A,B)** Representative immunohistochemistry images **(A)** of NeuN staining in the hippocampus of shMettl3-injected mice and quantifications **(B)** of relative NeuN positive cell numbers in GFP-positive area. n = 3, *F* = 0.626, t = 0.473, df = 4, *p* = 0.015, * *p*<0.05, by student’s t-test. **(C–E)** Representative images of the different cell markers by immunocytochemistry in hippocampus **(E)**, quantification **(C)** of the number of DCX^+^ cells and quantification **(D)** of the number of Iba1^+^ cells. Data are mean ± SD (*n* = 3) (DCX: *F* = 11.673, *t* = −0.029, df = 4, *p* = 0.978; Iba1: *F* = 5.032, *t* = 4.671, df = 4, *p* = 0.01) *p < 0.05, ns not significant, unpaired student’s *t*-test. **(F)** RT-qPCR analysis of PD-1/PD-L1 pathway-related genes in hippocampus after Mettl3 knockdown. Data are mean ± SD (*n* = 3, *Pdcd1*: *F* = 7.636, *t* = −13.255, df = 4, *p*<0.000; *Myd88*: *F* = 2.753, t = −12. 675, df = 4, *p*<0.000; *Ptpn6*: *F* = 2.032, t = −10.086, df = 4, *p* = 0.001) *p<0.05, ***p<0.001, student’s *t*-test.

As neuron in the hippocampus is the basis of spatial cognition, we observed the effect of *Mettl3* knockdown on neurons. NeuN protein is strongly expressed in the early stages of neuronal differentiation, and we found that it was reduced in shMETTL3-injected mice compared with controls, especially in the pyramidal neurons. This suggesting pyramidal neurons are more sensitive to the change of Mettl3. (Figures 8A,B; Supplementary Table S2). Interestingly, in contrast to the significant loss of neurons seen in shMETTL3-injected mice, there were no changes in immature neurons, as evidenced by our analysis of DCX-positive neurons (Figures 8C–E; Supplementary Table S2). Given the possible effect of methylation on microglia described above, we analyzed changes in microglia in shMETTL3-injected mice hippocampus. Iba1 immunostaining showed that microglia were significantly reduced in the region of METTL3 knockdown. This suggested that *Mettl3* knockdown in the hippocampus induces neurodegeneration and reduction of number in microglia, which may underlie the spatial cognitive deficits seen in shMETTL3-injected mice.

We found that *PD-1*(*Pdcd1*), *Myd88*, and *Ptpn6* were upregulated in the hippocampus of 64-week-old mice at both the methylation and transcriptional levels. Understanding the regulation of these genes may facilitate the treatment of hippocampal aging-related cognitive impairment. Therefore, we investigated whether METTL3 plays a regulatory role in the expression of these three genes. Knockdown of METTL3 increased the mRNA levels of *PD-1*, *Myd88*, and *Ptpn6* (Figure 8F; Supplementary Table S2). This finding suggests that METTL3 affects the expression of *PD-1*, *Myd88*, and *Ptpn6* in the PD-1/PD-L1 pathway.

## 4. Discussion

It is well known that m^6^A RNA methylation plays an important role in brain development and aging. This study revealed the dynamics of m^6^A methylation in the postnatal mouse hippocampus through analyses of P10, 11-week-old, and 64-week-old mice. m^6^A methylation was found to regulate hippocampal function and act on different hippocampus cells at different timepoints. Moreover, m^6^A contributed to hippocampal-related cognitive dysfunction in the aging (64-week-old) hippocampus via the PD-1/PD-L1 pathway.

The mouse hippocampus develops rapidly after birth, in terms of both volume and structure, and matures during adulthood (at around 12 weeks of age). However, physiological and structural changes in neurons lead to age-related cognitive decline. Although methylation modifications, of which m^6^A is one of the most common, are highly enriched in the mammalian brain (Meyer et al., 2012), the importance of this epigenetic marker in brain development and aging is only just beginning to be appreciated. mRNA modifications persist throughout the mammalian lifespan (McMahon et al., 2021). We found that m^6^A regulates hippocampal function across the postnatal lifespan. At P10, which is a period of rapid postnatal hippocampal development, we found that DMGs and specifically methylated genes were more strongly correlated with neuronal functions, such as synaptic transmission and axonogenesis, compared with 11- and 64-week-old mice. These results are consistent with a previous study (Ma et al., 2018). Meyer et al. found that m^6^A levels are developmentally regulated, and that methylated transcripts are highly biased toward neuronal genes and functions in rodents (Meyer et al., 2012). Furthermore, our bioinformatics analysis showed that both temporal-specific and differentially methylated m^6^A mRNA was enriched in neuronal stem cell-specific genes at the P10 stage; this suggests that m^6^A RNA methylation during hippocampal development contributes to neuronal development and thus improves cognitive function. Once hippocampal development reached a stable stage (at 11 weeks), TSMRs were predominantly enriched in protein localization, which is fundamental for life. Our data suggest that dynamic regulation of m^6^A during hippocampal development (P10) and adulthood (11 weeks) ensures normal development. The dynamic regulation of m^6^A modification in the brain continues throughout the lifespan. There is increasing evidence that m^6^A methylation not only regulates development but is also involved in important changes that occur during aging and mediates the onset and development of many age-related diseases (Rubio et al., 2018; Wu et al., 2020; Chen et al., 2021; Yen and Chen, 2021). We identified differentially methylated transcripts in three stages of mouse hippocampal development. Genes with altered m^6^A levels in the P10 and 11-week-old groups exhibited extensive and profound changes in expression at the transcriptional level. In contrast, at 64 weeks, hypermethylated transcripts had a greater effect on the expression of mRNA levels than hypomethylated and consistently methylated genes; these genes are involved in oxidative phosphorylation. We hypothesize that gene expression stabilizes in old age, and that genes showing simultaneous changes in methylation and transcription levels are highly correlated with specific changes seen with aging. Furthermore, unlike at the P10 stage, m^6^A was predominantly enriched in neurogenesis-related cells and astrocytes in the 11-week-old groups, while transcripts showing differential methylation in the 64-week-old hippocampus were predominantly enriched in microglia and T cells. Microglia are critical for the regulation and maintenance of neuronal function (McMahon et al., 2021); they are the primary innate immune cells of the brain and the first cells to respond to various stimuli. Recent studies showed that microglia may mediate synaptic loss during the course of AD (Bartels et al., 2020), and that synaptic loss is strongly associated with cognitive impairment. Microglia activation can be observed in neurodegenerative diseases and brain aging, but the signaling pathways and mechanisms involved are unknown. In our study, methylation modifications were significantly enriched in microglia in the aged hippocampus.

The PD-1/PD-L1 signaling pathway has been extensively studied in the context of tumor diseases, and blockade of the pathway with checkpoint inhibitors has promise for the treatment of many forms of cancer (Sharma and Allison, 2015). Studies on the PD-1/PD-L1 pathway in non-tumor cells found that increased activity of the PD-1/PD-L1 pathway is associated with aging, including of T cells exhibiting cellular senescence (Lages et al., 2010). In AD patients and mouse models, PD-1 expression was upregulated in astrocytes and microglia (Kummer et al., 2021). In addition, a recent study found that enhanced expression of PD-1 was highly correlated with reduced kidney function in aging humans (Pippin et al., 2022). In our study, PD-1/PD-L1 pathway activity was significantly enhanced in the aging (64-week-old) hippocampus at both the methylation and transcriptional levels. However, the hippocampal expression level in the other two stages was very low, especially in the P10 stage. To our knowledge, this is first study of PD-1 expression in different life stages in the mouse. Based on the above studies, we speculate that the PD-1/PD-L1 pathway may be important in age-related hippocampal dysfunction, and is likely regulated by m^6^A. The upregulated genes (*Pdcd1, Myd88 and Ptpn6*) in the 64-week-old hippocampus were highly m^6^A-methylated. This corroborates previous studies reporting that m^6^A methylation promotes gene expression (Liu et al., 2019; Xu et al., 2021). However, knockdown of METTL3 was accompanied by upregulation of *Pdcd1, Myd88*, and *Ptpn6* mRNA expression. m^6^A regulation of gene transcription is a complex process; a given methylase may upregulate, downregulate, or even have no effect on the same gene in different signaling pathways and diseases. Although the effect of METTL3 on PD-1 needs to be confirmed by further studies, targeting METTL3 could help prevent cognitive impairment related to hippocampal aging.

The dynamic m^6^A modification in the mouse hippocampus is important in various biological processes accomplished by m^6^A methyltransferases. In our study, we identified the methylated mRNA transferase METTL3, the expression of which varies dynamically across the lifespan. The mRNA and protein levels of METTL3 were higher in the 11-week-old hippocampus compared with the other two stages. The behavioral and morphological changes observed during transient knockdown of METTL3 in 11-week-old-mice confirmed that dysregulation thereof affects hippocampal-related cognitive functions. METTL3 is not the only m^6^A methylase that affects hippocampal related cognitive functions; YTHDF1, the “reader” of m^6^A, facilitates hippocampus-dependent learning and memory (Shi et al., 2018). These findings suggest that m^6^A plays an important role in hippocampal-related cognition. In addition, we found that *Mettl3* expression was decreased in the hippocampus of 64-week-old mice. In a study of the role of *Mettl3* in age-related neurodegenerative diseases, *Mettl3* levels were reduced in the brains of patients with mild cognitive impairment, which is the prodromal phase of AD, as well as in AD patients (Zhang et al., 2018; Huang et al., 2020). Furthermore, *Mettl3* was associated with synaptic loss and neuronal reduction in the hippocampus of AD mice (Zhang et al., 2018; Zhao et al., 2021). This is consistent with our finding of *Mettl3*-induced cognitive dysfunction, where dysregulation of *Mettl3* reduced new mature neurons in 11-week-old mice. In addition to lower levels of *Mettl3* expression in the 64-week-old hippocampus, *Mettl3* is also expressed at lower levels in the P10 stage. A role for *Mettl3* in development has been demonstrated: knockout of *Mettl3* in mice led to early embryonic lethality (Geula et al., 2015), and *Mettl3* regulates the development of the mammalian cerebellum through its effects on RNA half-life and splicing events (Wang et al., 2018). We detected relatively weak *Mettl3* signals in the lateral hippocampal dentate gyrus of P10 mice. At the 11-week-old stage, in which the granule cell layer had formed, METTL3 protein expression increased significantly. Knockdown of METTL3 resulted in a decrease in the number of new mature neurons in the hippocampus of 11-week-old mice. We speculate that enhanced expression of *Mettl3* is a key event in the formation of mature granule neurons. Therefore, we suggest that the relatively low expression of METTL3 in P10 is associated with the low number of mature granule cells. Interestingly, the effect of METTL3 knockdown on neurons was limited to mature ones; neural progenitor cells were not affected. Microglia alterations may be the underlying mechanism of age-related decreases in adult neurogenesis (Terreros-Roncal et al., 2021). In our study, knockdown of METTL3 decreased microglia expression, suggesting that there is a close relationship between m^6^A methylation and microglia. However, whether methylation is involved in microglial cell activation needs to be further explored; this may improve our understanding of the pathogenesis of age-related cognitive impairment in the hippocampus.

In summary, our study provides insight into the changes in m^6^A methylation modifications occurring in the mouse hippocampus throughout the postnatal lifespan. The results indicate that RNA methylation modifications in the hippocampus of aged mice affect spatial cognitive function through the PD-1/PD-L1 pathway. Furthermore, we provide *in vivo* evidence that METTL3 can cause aberrant expression of *Pdcd1* at the transcriptional level. These results provide a platform for elucidation of the molecular mechanisms of hippocampal aging and could lead to new interventions and treatments for age-related diseases.

## Data availability statement

The datasets presented in this study can be found in online repositories. The names of the repository/repositories and accession number(s) can be found in the article/Supplementary material.

## Ethics statement

The animal study was reviewed and approved by Capital Medical University’s Animal Care and Use Committee.

## Author contributions

WH designed the study and drafted the paper. HX, YZ, and PP carried out the mouse study. XZ revised the manuscript. SW and ZW contributed to the data acquisition, interpretation, and discussion. All authors contributed to the article and approved the submitted version.

## Funding

This work was supported by the National Natural Science Foundation Projects (81670904 and 81971390), Research Foundation of Capital Institute of Pediatrics (FX-2020-05, CXYJ-2-21-09), public service development, and reform pilot project of Beijing Medical Research Institute (BMR2021-3). Beijing Hospitals Authority’s Ascent Plan (DFL20221102).

## Conflict of interest

The authors declare that the research was conducted in the absence of any commercial or financial relationships that could be construed as a potential conflict of interest.

## Publisher’s note

All claims expressed in this article are solely those of the authors and do not necessarily represent those of their affiliated organizations, or those of the publisher, the editors and the reviewers. Any product that may be evaluated in this article, or claim that may be made by its manufacturer, is not guaranteed or endorsed by the publisher.

## Footnotes

^1^ https://software.broadinstitute.org/software/igv/
