# Supplementary figures and images for: N6-methyladenosine participates in mouse hippocampus neurodegeneration via PD-1/PD-L1 pathway
Source: Front Neurosci. 2023 May 10;17:1145092. doi: 10.3389/fnins.2023.1145092 (PMC10206131; doi:10.3389/fnins.2023.1145092)

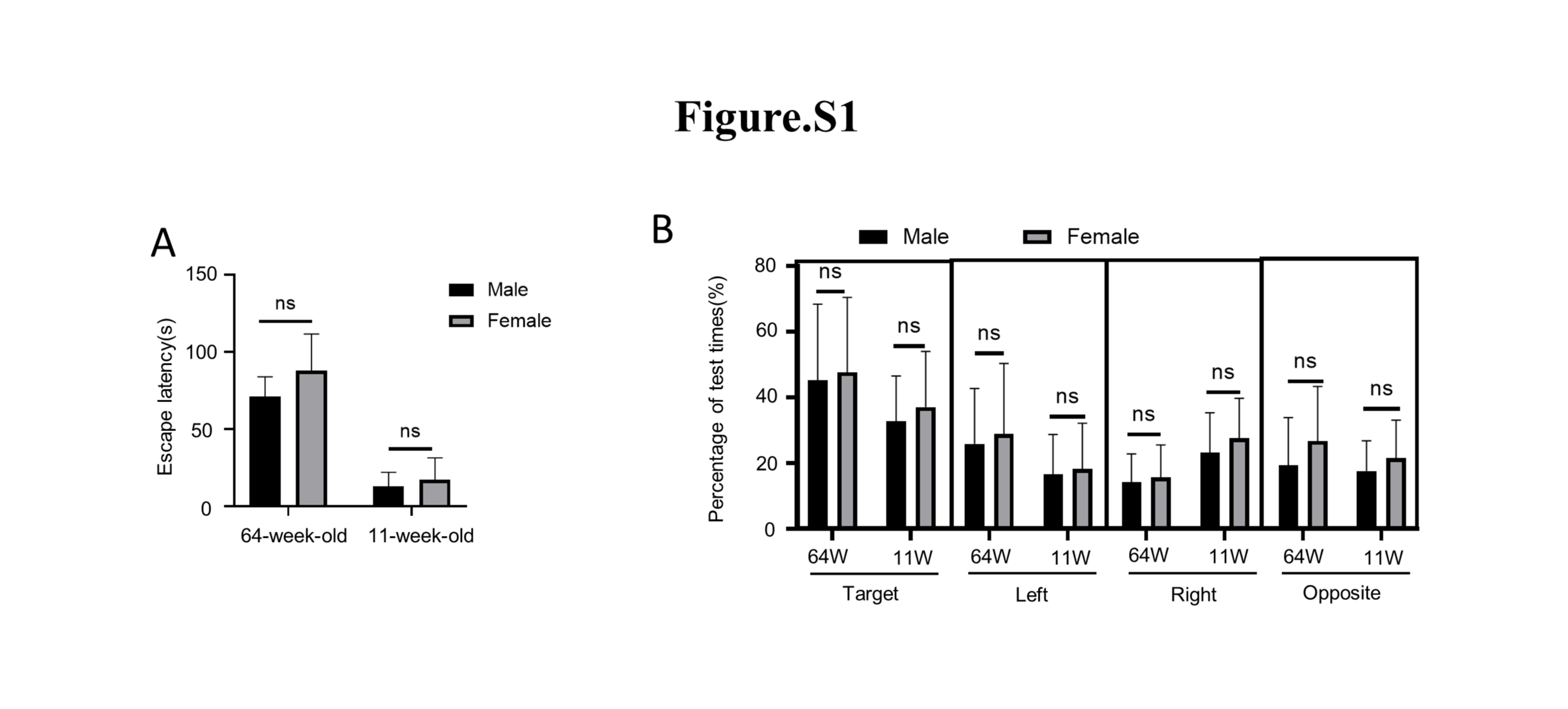

Supplement: Supplementary file 1 [file Image_1.TIF]

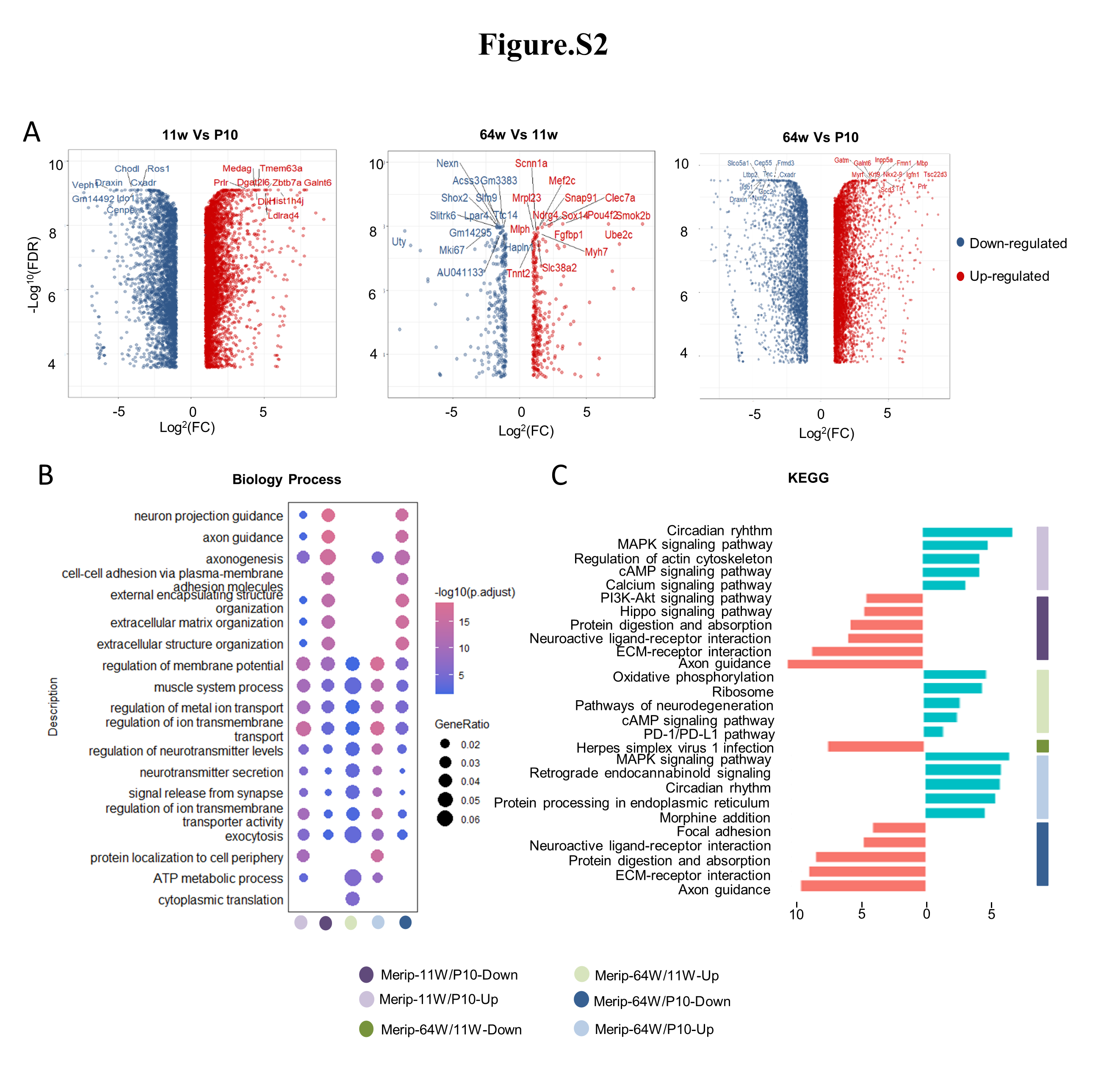

Supplement: Supplementary file 2 [file Image_2.TIF]

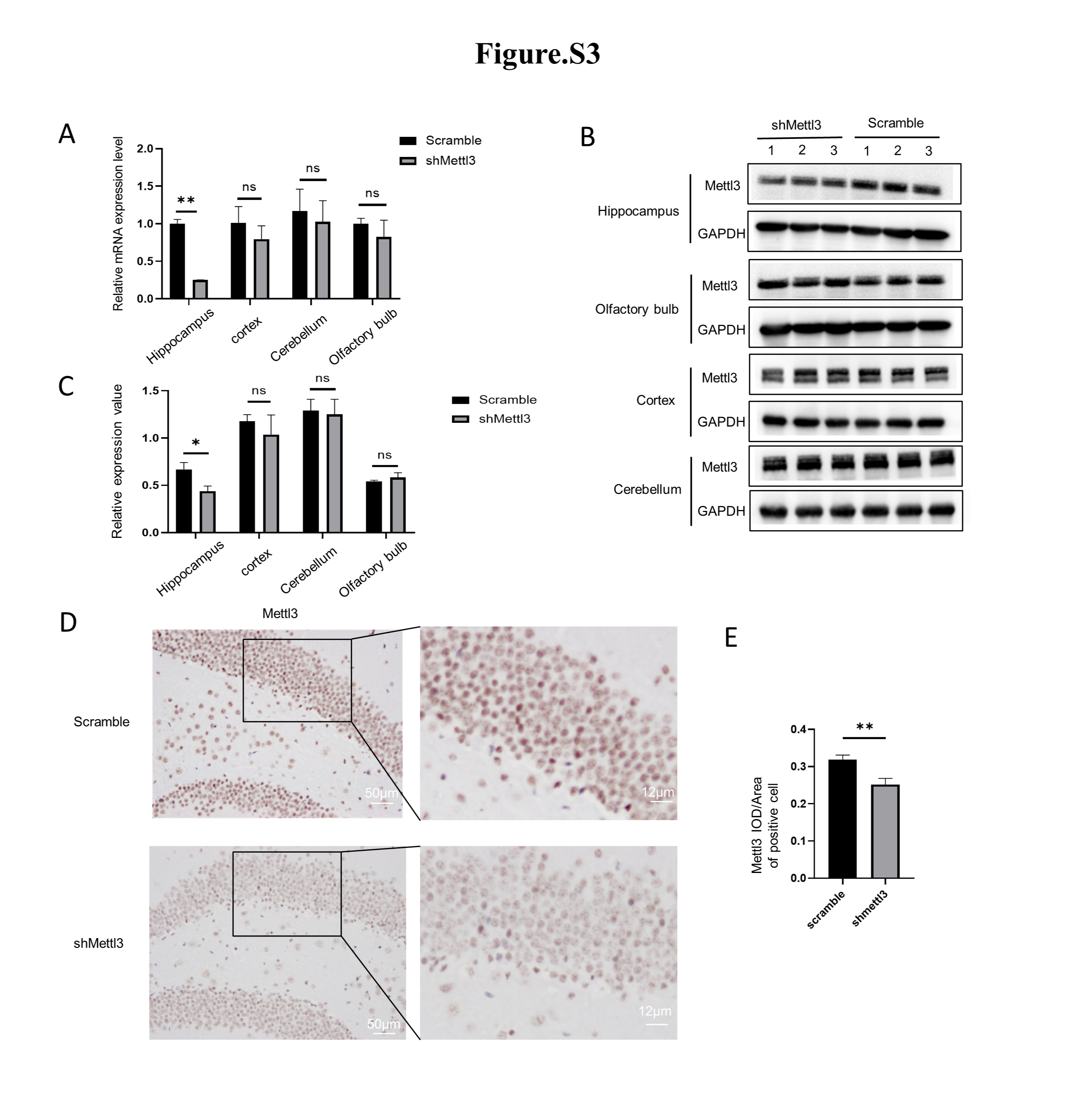

Supplement: Supplementary file 3 [file Image_3.TIF]
